# A Satisfactory Financial Position

**Published:** 1916-07-29

**Authors:** 


### LEICESTER ROYAL INFIRMARY.

### A Satisfactory Financial Position.

At the half-yearly meeting of the Governors, the new chairman, Mr. T. Fielding Johnson, jun., presided, and important administrative changes and substantial progress in the affairs of the hospital were reported. Satisfaction was expressed at the liquidation of the outstanding debt on the Children's Hospital Building Fund, and that upwards of £4,000 had been raised for the construction of the new pathological department and new post-mortem room, which would be erected immediately a favourable opportunity arose. A further urgent need was represented in the provision of enlarged accommodation for the increased nursing staff, and it was estimated that the twenty additional bedrooms which were immediately required would involve an expenditure of £3,000. One thousand eight hundred and seventy-one admissions to the civil wards of the institution and 106 to the military wards were recorded during the six months. The average daily number of beds occupied was 240.

The income and expenditure account was satisfactory. The receipts were £15,157, of which £6,200 arose from special and adventitious sums, legacies, and special income producing £4,378, and Government grants for the maintenance of soldiers £1,820. The expenditure for the six months was £15,300, which, added to the deficit on 1915 accounts, £2,953, made a total of £18,250. The increased cost of maintenance due to enhanced prices was 35 per cent. over that of 1912, a pre-war period in which the work accomplished was for practical purposes comparable.

Complimentary reference was made to the work, under difficult conditions, of the honorary staff, the resident doctors, Miss Vincent (matron) and her nursing staff, and the house governor and secretary of the institution. The duty which Mr. Alfred Corah (High Sheriff) had undertaken in securing new and increased annual subscriptions was heartily eulogised. He had been assisted in this task by Mr. B. W. Russell, Mr. F. S. Brice, and Mr. Duncan Henderson, and reported that he had secured about fifty new and increased subscriptions of 50 guineas each and about twenty of 25 guineas each. The work was being continued, and he hoped as a result the annual subscription list would be raised to at least £10,000.

A proposal had been set on foot and was heartily supported by the administrative and military officials of the county for the endowment of a bed in the institution to the memory of Viscount Kitchener, whose father, Lieutenant-Colonel H. H. Kitchener, resided for many years in the county. The passing of time brought new needs to the institution, and it behoved the governors to look well ahead to the period when the present terrible war was over. There would be many cases of crippled and disabled soldiers seeking medical and surgical treatment. Some such had already presented themselves at the institution on their discharge as medically unfit for further service. The Board did not doubt that the residents of Leicester and Leicestershire would by their continuous and increased support make the Royal Infirmary efficient and adequate to meet present and future needs.

